# Effect of pomegranate (*Punica granatum L*) peel powder meal dietary supplementation on antioxidant status and quality of breast meat in broilers

**DOI:** 10.1016/j.heliyon.2020.e05709

**Published:** 2020-12-16

**Authors:** Eunice A. Akuru, Chika E. Oyeagu, Thando C. Mpendulo, Fanie Rautenbach, Oluwafemi O. Oguntibeju

**Affiliations:** a Department of Livestock and Pasture Science, University of Fort Hare, Private Bag X1314, Alice, 5700, Eastern Cape, South Africa; b Department of Animal Science, University of Nigeria, Nsukka, 410001, Nigeria; c Department of Agriculture, Faculty of Applied Sciences, Cape Peninsula University of Technology, Wellington Campus, Private Bag X8, Wellington, 7654, Western Cape, South Africa; d Oxidative Stress Research Centre, Department of Biomedical Sciences, Cape Peninsula University of Technology, P.O. Box 1906, Bellville, 7535, South Africa

**Keywords:** Meat quality, Antioxidant capacity, Pomegranate peel, Diets, Free radicals, Food quality, Nutrition, Animal science, Agricultural technology, Organic farming

## Abstract

This study examined the antioxidant status and quality of breast meat in broiler birds fed diets supplemented with pomegranate peel powder meal (PPPM). During the 35-d feeding trial, broiler birds were fed six experimental diets: diet with 0% additives (negative control; NEGCON); diet with α-Tocopherol acetate at 200 g/tonne (positive control; POSCON); and four levels (2, 4, 6 and 8 g/kg) of PPPM, designated as PPPM_2_, PPPM_4_, PPPM_6_, and PPPM_8_. Breast muscle pH was determined 15mins and 24hrs postmortem. The breast muscles were then stored at 4 °C to determine shelf-life attributes (pH, colour, hue angle, and chroma) for 16 days. Meat from the 8 g/kg PPPM had the highest thawing loss, whereas cooking loss was lowest at 2 g/kg PPPM inclusion. The meat of birds fed 2 g/kg and 4 g/kg PPPM had the highest (P<0.05) ability to scavenge the ABTS [(2, 2-azinobis (3ethylbenzothiazoline-6 sulfonic acid))] radical cation (ABTS^+^), whereas, catalase activity was increased at 8 g/kg PPPM. The results obtained in this study indicate that 2 g/kg supplementation of pomegranate peel powder meal significantly improved the water-binding capacity of broiler breast meat, owing to the reduced cooking loss of the meat, and meat from the PPPM_2_ (2 g/kg) group had the highest ability to scavenge ABTS.

## 1 Introduction

The system of genetic selection employed in the broiler industry with a focus on important traits like fast growth rate and high muscle yield has led to increased incidence of metabolic muscle myopathies such as wooden breast and white striping, purge in meat, and lipid oxidation (Petracci et al., 2015). The oxidative damage of lipids or fats is usually associated with a marked deterioration in the organoleptic, shelf-life, and nutritive properties of chicken meat (Taslimi et al., 2018). This damage to lipids consequently decreases the acceptance of the affected meat products by consumers (Falowo et al., 2014), resulting in economic loss. Broiler meat contains polyunsaturated fatty acids (PUFAs) as a normal form of fat (Adamski et al., 2017). However, supplementing broiler diets with high levels of PUFAs causes the meat to become increasingly susceptible to rancidity due to lipid oxidation (Estevez, 2015). Oxidation of PUFAs results in the production of harmful chemicals such as hydro-peroxides, which are further decomposed into short-chain aldehydes, ketones, and other oxygenated compounds which exert a harmful effect on the synthesis and metabolism of lipids, pigments, proteins, carbohydrates, and vitamins (Lobo et al., 2010; Shukla et al., 2011). These free radicals are responsible for the mutagenic, carcinogenic, and aging processes in biological systems (Giustarini et al., 2009; Ighodaro and Akinloye, 2018).

Every living cell has endogenous defense mechanisms that protect it against the harmful effects of free radicals that result in oxidative damage, and ultimately, oxidative stress. These protective mechanisms function properly owing to the activities of enzymatic and non-enzymatic antioxidants like superoxide dismutase, catalase, glutathione peroxidase, transferrin, etc (Halliwell and Gutteridge, 2007). However, the production of free radicals above endogenous protection, due to diet deficiencies and breed differences in animals, coupled with inappropriate pre-slaughter handling procedures, exposes animals to oxidative stress (Xing et al., 2019). Hence, there is a need to overcome this deficiency through dietary antioxidant intervention (Malireddy et al., 2012). Natural antioxidants derived from plants have various immune-enhancing effects due to their polyphenol contents (Krawczk, 2019). Natural antioxidant compounds also can enhance the synthesis and activity of antioxidant enzymes and PUFAs in animals' tissue (Shalaby and Shanab, 2013; Amaral et al., 2018).

Pomegranate (*Punica granatum L*) is an important ornamental plant that belongs to the Lythraceae family and is extensively grown in many parts of the world, including South Africa (Arendse et al., 2017a, b). Pomegranate peel is the inedible portion of the pomegranate plant that makes up about 50% of the total fruit weight (Fawole and Opara, 2016). Pomegranate peel has antioxidant, antimicrobial, hypoglycemic, hypolipidemic, non-cytotoxic, hepatoprotective, and anti-inflammatory properties (Rajput et al., 2011; Pagliarulo et al., 2016). Pomegranate peel improves meat's oxidative stability owing to its rich natural antioxidant content (Descalzo and Sancho, 2008). This improvement is due to its ability to effectively scavenge the active forms of reactive oxygen species (ROS), which are involved in the initiation and progressive phases of oxidation (Rajani et al., 2011). The scavenging ability of pomegranate peel is attributed to its content of various compounds such as the hydrolyzable tannins, including ellagitannin, gallotannins, and gallagyl esters like punicalagin, punocaliin, and pedunculagin (Madrigal-Carballo et al., 2009). There are also considerable amounts of flavonoids, catechins, ellagic acid, flavonones, flavones, anthocyanidins, and several polyphenols found in pomegranate peel (Naveena et al., 2008).

Over the years, the extracts of pomegranate fruit peel have had positive effects on the meat quality and antioxidant capacity of broiler meat (Chandralekha et al., 2012; Saleh et al., 2017a; b; Sarica and Urkmez, 2018; Kishawy et al., 2019; Sharifian et al., 2019). Vitamin E (α-tocopherol acetate) is a fat-soluble radical scavenging supplement that can also delay lipid oxidative processes in meat and its associated products (Karami et al., 2010; Li and Liu, 2012). In view of, this study was carried out on the premise that minimal literature exists on the use of pomegranate peel powder in improving the meat quality and antioxidant potential of broiler breast meat. More so, it is noteworthy that the South African grown "Wonderful" pomegranate variety used in this study has not been previously utilized in broiler nutrition. Therefore, the present study was conducted to investigate the effects of dietary pomegranate peel powder meal supplementation on the quality and antioxidant enzyme capacity of broiler breast meat.

## 2 Materials and methods

### 2.1 Ethical statement

Ethical approval for the study was sought and obtained from the Animal Research Ethics Committee of the University of Fort Hare, Alice (Ethical clearance number: MUC061SAKU01). Permission to conduct research was also obtained in Section 20 of the Animal Diseases Act, 1984 from the Department of Agriculture, Forestry and Fisheries (DAFF) South Africa with reference number: 12/11/1/4. Based on the ethical approvals that were granted, the present study complies with all ethical regulations.

### 2.2 Study location and ingredients sources

The 35-d experimental feeding trial was carried out at the poultry section of the Fort Cox College of Agriculture and Forestry Training institute located at Middledrift, Eastern Cape, South Africa, on the following coordinates 32.46^o^S, 27.02^o^E. Fresh pomegranate peels were supplied by the Post-harvest Research Center, Stellenbosch University. The vitamin E (α-tocopherol acetate) was procured from Merck (Pty) Ltd Modderfontein, South Africa. All other feed ingredients were procured from Monti Feeds (East London, South Africa).

### 2.3 Peel collection and preparation

Fresh pomegranate peels ('Wonderful' variety) were obtained from the Post-harvest research center of Stellenbosch University and dried as described by Mphahlele et al. (2016), with slight modifications. The peels were put in clean trays and weighed. The peels were dried at 60 °C in an oven (Model No. 072160, Prolab instrument, Sep Sci., South Africa). During drying, a change in weight was recorded using a digital balance (ML3002.E, Mettler Toledo, Switzerland) at an hourly interval. The moisture content of the peels was determined by drying peels to reach equilibrium, i.e., until there were no more weight changes. Usually, moisture content of 8% (wet basis) is reached after 22 h. The peels were removed from the oven, put in a polymer bag, and stored at 5 °C until use. Afterward, the dried peels were milled into powder using a milling machine to pass through a 0.15-mm sieve. The ground power was stored at -20 °C until needed for extraction, analysis, and feeding trial. The proximate contents (Table 1) of the pomegranate peel powder (PPP) were determined according to the methods described by the Association of Analytical Chemists (AOAC, 2000). The mineral composition of the PPP (Table 1) was determined using the guidelines of AgriLASA (1998).

**Table 1:** Proximate and mineral composition of pomegranate peel powder.

Parameter	Quantity
Crude protein (%)	2.17
Moisture (%)	6.67
Ash (%)	4.06
Ether extract (%)	6.54
Acid detergent fibre (%)	26.90
Neutral detergent fibre (%)	34.50
Calcium (%)	1.05
Phosphorus (%)	1.24
Potassium (%)	1.82
Magnesium (%)	0.55
Sodium (%)	0.31
Copper (mg/kg)	37.00
Iron (mg/kg)	279.00
Zinc (mg/kg)	15.10
Manganese (mg/kg)	15.70

### 2.4 Preparation of peel extracts

About 2.5g of dried pomegranate peel powder was extracted using 80mL ethanol solvent under shaking for 48 h. The crude extract was filtered under pressure using a Buchner funnel and Whatman No. 1 filter paper. The filtrate was then concentrated under vacuum at 30 °C using a high capacity rotary evaporator (Strike 202 Steroglass, Italy). A lyophilizer (Vir Tis benchtop K, Vir Tis Co, Gardiner, NY) was used to dry the ethanol-free extract, after which the dried samples were stored at -70 °C until needed for analysis.

### 2.5 Determination of phytochemical and antioxidant contents of pomegranate peel extract

The total polyphenol of the extract of pomegranate peel powder (PPP) was determined using the Folin Ciocalteu's phenol reagent based on the methods of Singleton et al. (1998). The total antioxidant capacity of the extract was determined using the oxygen Radical Absorbance Capacity (ORAC) assay based on the fluorometric method described by Ou et al. (2001). The ABTS [(2, 2-azinobis (3ethylbenzothiazoline-6 sulfonic acid))] scavenging ability of the extract was analyzed by the Trolox Equivalent Antioxidant Capacity (TEAC) using the method described by Re et al. (1999). The ferric reducing antioxidant power (FRAP) assay of the extract was determined using the method described by Benzie and Strain (1996). The antioxidant and phytochemical composition of the pomegranate peel extract is shown in Table 2.

**Table 2:** Phytochemical and antioxidant composition of pomegranate peel extract.

Parameter	Concentration
ORAC (μmol TE/g)	1006.29
FRAP (μmol AAE/g)	696.51
ABTS^+^ (μmol TE/g)	507.93
Polyphenols (mg GAE/g)	143.98
Flavonols (mg QE/g)	16.75
Flavanols (mg CE/g)	N.D.

TE; Trolox equivalents, AAE; Ascorbic acid equivalents, GAE; gallic acid equivalents, QE; Quecertin equivalents, CE; Catechin equivalents, N.D; none detected.

### 2.6 Dietary treatments

The study consisted of two experimental (starter and grower-finisher) phases, during which six isonitrogenous and isocaloric diets (Table 3) were formulated to meet the dietary requirements of the broiler birds (National Research Council, 2008). The experimental diets were designated as: T_1_-control diet with 0% additives (negative control; NEGCON); T_2_-control diet supplemented with α-tocopherol acetate at 200g per ton (positive control; POSOCON): T_3_-control diet supplemented with 2 g/kg PPPM (PPPM_2_); T_4_-control diet supplemented with 4 g/kg PPPM (PPPM_4_); T_5_-control diet supplemented with 6 g/kg PPPM (PPPM_6_): T_6_-control diet supplemented with 8 g/kg PPPM (PPPM_8_). The proximate composition of the experimental diets was determined based on the methods of the Association of Analytical Association of Official Analytical Chemists (2000). The concentrations of acid detergent fibre and neutral detergent fibre (Table 3) in the diets were determined according to the methods described by Van Soest *et al.* (1991). The mineral composition of the diets (Table 3) was determined based on the guidelines of AgriLASA (1998).

**Table 3:** Ingredients and nutrient composition of basal diet.

Ingredients	Starter (0–21days)	Grower-finisher (22–35days)
Maize	48.84	58.00
Soybean full fat	28.50	36.78
Soybean meal (CP 44.0%)	13.25	-
Fishmeal 65	4.00	-
L-lysine Hcl	0.15	0.13
DL-methionine	0.40	0.32
L-threonine	0.16	0.05
a Vit + min premix	0.15	0.15
Limestone	1.46	1.40
Salt	0.20	0.25
Monocalcium phosphate	1.23	1.32
Sodium bicarbonate	0.16	0.10
Sunflower oil	1.50	0.15
**Calculated composition (%)**		
ME (MJ/kg)	13.18	13.81
Crude protein	24.07	19.38
Crude fibre	4.56	3.34
Ether extract	5.54	6.86
Calcium	1.03	1.01
Available phosphorous	0.44	0.37
Lysine	1.44	1.06
Threonine	0.89	0.70
Tryptophan	0.28	0.21
**Analyzed composition (%)**		
Crude protein	23.24	20.05
Ash	5.34	5.16
Ether extract	8.89	8.70
Acid detergent fibre (ADF)	4.63	4.86
Neutral detergent fibre (NDF)	14.44	20.09
Calcium (%)	1.41	1.36
Phosphorus (%)	0.78	1.23

a Vitamin + mineral premix provided (per kg of feed): 8160 IU vit A, 1700 IU vitamin D_3,_ 30.6 IU vitamin E, 2.7mg vitamin K_3_ 205mg vitamin B_1_, 2.03mg vitamin B_2,_ 27.2mg niacin, 10.2mg calcium pentothenate, 2.02mg vitamin B12, 4.1mg vitamin B6, 1.7mg folic acid, 0.068mg biotin, 120mg ronozyme P500, 350mg choline, 0.08mg I, 0.34 mg Co, 0.2mg Se, 70mg Mn, 70mg Zn, 6 mg C and 50mg Fe.

### 2.7 Experimental animals and management

A total of 432 day-old Cobb-500 broiler chicks were used for the study. Upon arrival, an anti-stress (stress pack) vitamin was administered to the chicks via clean water at 100 g/50 L (according to manufacturer's recommendation) to help them combat travel stress, boost their appetite and energy supply. This process was repeated weekly after weighing the birds. After that, the chicks were individually weighed and randomly assigned to six dietary treatments, with four replications of 18 birds per replicate. The six experimental diets were formulated to meet the nutrient requirements of birds at the starter (0-21d) and grower-finisher (22-35d) phases, based on the primary breeder's recommendations. The temperature of the broiler house at the start of the feeding trial was set at 35 °C, and thereafter, reduced gradually by 2–3 °C weekly until it reached 22 °C in the 5th week. A-24 h lighting regimen per day was provided for the first 72 h to stimulate feeding and drinking in the young chicks. The lighting was reduced to 23 h per day by the end of the first week (day 7) of life. After that, a step-down lighting program was followed until slaughter. Artificial bulbs were used as the source of light. The birds were given the Gumboro disease vaccine at days 7 and 14 of the feeding trial, while, New Castle disease vaccine was administered on 21 and 28 days of age. Dietary treatments and clean water was supplied to the birds *ad libitum* during the five weeks of the feeding trial.

### 2.8 Slaughter of birds and collection of samples for meat quality analysis

On the 35th day of the feeding trial, 24 birds (one bird per replicate) were randomly selected around the same mean weight of birds per pen. The birds were fasted for 6 h and humanely slaughtered by cervical dislocation after being electrically stunned at 70V. Samples of breast (Pectoralis major) meat from each bird were stored at 4 °C for 16 days and used to determine meat quality and antioxidant enzyme capacity.

### 2.9 Evaluation of meat quality parameters

#### 2.9.1 Determination of thawing loss

The initial weights of frozen breast meat samples were recorded (frozen weight), and then the meat was allowed to defrost for 12 h at room temperature. Upon defrosting, the meat samples were weighed again (defrosted weight). Thawing loss percentage was calculated by subtracting the frozen value from the defrosted weight and expressing it as a proportion of the frozen weight.

#### 2.9.2 Determination of cooking loss

The initial weights of meat samples that had been allowed to defrost at room temperature were recorded (uncooked weight). Afterward, samples were carefully loaded into an oven set at 120 °C for 35 min. The samples were removed, allowed to cool for 10–15 min, and then the cooked weight was recorded. Cooking loss was determined by subtracting the already defrosted uncooked weight from the cooked weight and expressing it as a percentage of the raw weight of the meat samples.

#### 2.9.3 Determination of meat tenderness

The same cooked meat samples were used to determine meat tenderness using the shear force apparatus. Sub-samples from the cooked breast muscles were sheared in a direction perpendicular to the direction of the fibre, using a Warner - Bratzler shear device mounted on a Universal Instron machine (crosshead speed of 300 mm/min).

### 2.10 Effect of extended storage of meat on pH and color

#### 2.10.1 Meat pH measurements

The initial pH (pH_i_) of broiler breast meat was determined 15 min after slaughter using a temperature compensating pH meter equipped with an electrode (CRISON pH 25, CRISON Instruments, S.A. Allela, Spain). Standard buffers (10.0, 7.0, and 4.0) were used to calibrate the pH meter. Carcasses were packed in transparent storage bags and hung in cold storage for 24 h at 4 °C. Following the 24hr storage period, the ultimate pH (pH_u_) was determined. Subsequent pH readings were recorded every morning for 16 consecutive days.

#### 2.10.2 Meat colour measurements

The instrumental color (L∗ = lightness, a∗ = redness, b∗ = yellowness) indices were measured 24 h postmortem on the ventral side of the right breast fillet with a Minolta color-guide (BYK-Gardner GmbH, Gerestried, Germany), with illuminant D65 and a 2.54-cm aperture. The readings were recorded in triplicates, and the averages were calculated and used for statistical analysis. Subsequent color readings were recorded every morning for 16 consecutive days. Hue angle (indicates the angle at which a vector radiates into the red-yellow quadrant), and the chroma (measures color saturation) was calculated using individual a∗ and b∗ values as shown in Eqs. (1) and (2) below:

(1) Hue angle (o) = tan^-1^ (b^∗^)/(a^∗^)

(2) Chroma (C) = (a^∗2^ + b^2∗^) 0.5

#### 2.10.3 Preparation of breast meat homogenates

5.0 g sample of the Pectoralis major was homogenized on ice in 10 volumes of ice-cold buffer (50mM sodium phosphate buffer with Triton - X, pH 7.5) with the aid of liquid nitrogen and tissue grind tube. The homogenates were centrifuged at 10000 ×g for 10 min at 4 °C. The resulting supernatants were collected, stored at - 80 °C, and used to determine antioxidant capacity and enzyme activity.

### 2.11 Meat antioxidant capacity and enzyme assays

Before analysis, sub-samples of breast meat samples from broilers were deproteinized with 0.5M perchloric acid (1:1, v/v) and centrifuged at 10000 ×g at 4 °C. The supernatant collected was a protein-free fraction stored at –80 °C until required for analysis (Robles-Sanchez et al., 2011). The total antioxidant capacity of broiler breast meat was determined using the TEAC assay described by Re et al. (1999). Briefly: 24 h before use, the ABTs solution was prepared and incubated in the dark pending when the analysis was performed. The solution was prepared by mixing 8mM ABTs salt with 140 mM potassium peroxodisulfate to encourage ABTS + radical cation formation. The ABTs solution was diluted in distilled water (1:20) to obtain an absorbance of 1.50 at 734nm. 10μL of the meat homogenate was added to the ABTS solution (275μL) in a 96-well transparent plate. The solution and the homogenate were properly mixed, and absorbance read at room temperature for 30 min in a Multiskan Spectrum plate reader. Trolox was used as standard, and the result was expressed as μM TE/g tissue. Superoxide dismutase (SOD) activity was determined based on the methods of Ellerby and Bredesen (2000), and the results expressed as U/mg protein. Catalase (CAT) activity in the breast meat samples was determined based on the methods of Aebi (1984), which involves measuring the rate of H_2_O_2_ decomposition at 232nm and also expressed as U/mg protein.

### 2.12 Statistical analyses

Data for the16-d shelf-life attributes (pH, colour, hue angle, and chroma), antioxidant capacity, and enzyme activity parameters were analyzed using the repeated measures analysis procedure of SAS (2009). The following statistical linear model was employed as shown in Eq. (3):

(3) $$Y_{ijk}\;=\;\mu \;+\;D_{i}+D_{j}+{\left(D\times D\right)}_{ij}\;+\;E_{ijk}$$

where: Y_ijk_ = dependent variable, μ = population mean, Di = effect of dietary treatments, D_j_ = effect of days (1, 2, 3…16), (D×D)_ij_ = effect of interaction between diets and days (1, 2, 3…16), E_ik_ = random error associated with observation ijk, assumed to be normally and independently distributed. Before analysis, all parameters were tested for normality using the NORMAL option in the Proc Univariate statement.

Data for postmortem physicochemical attributes of meat were subjected to analysis of variance for a Completely Randomized Design (CRD) using the General Linear Model Procedure of SAS (2009) with the statistical model shown in Eq. (4) below:

(4) Y_ij_ = μ + D_i_ + E_ij_

where Y_ij_ = Observed value of a dependent variable, μ = Overall population mean, Di = Effect of dietary treatments, and E_ij_ = Residual error associated with observation ij assumed to be normally and independently distributed. For all statistical tests, significance was declared at P<0.05.

## 3 Results

### 3.1 Physicochemical attributes of meat

The results on the physiochemical attributes of the breast meat of broiler birds fed diets supplemented with pomegranate peel powder meal (PPPM) are shown in Table 4. Meat from the 2, 6 and 8 g/kg PPPM (PPPM_2_, PPPM_6_ and PPPM_8_) had higher L∗ (lightness) value compared to the positive control (POSCON). Thawing loss was highest in meat from the PPPM_8_ group_,_ whereas cooking loss was lowest in meat from birds fed PPPM_2_ (2 g/kg PPPM) diet, when compared with the negative control (NEGCON) and POSCON diets.

**Table 4:** Physicochemical attributes of breast meat of broiler chickens fed dietary pomegranate peel powder meal supplementation.

Parameter	Treatments							
	NEGCON	POSCON	PPP_2g/kg_	PPP_4g/kg_	PPP_6g/kg_	PPP_8g/kg_	SEM	P-value
pH_15min_	5.52	5.57	5.51	5.59	5.43	5.49	0.03	0.69
pH_24hr_	5.67	5.69	5.65	5.69	5.64	5.76	0.02	0.89
L∗	51.14^a^	47.37^b^	50.71^a^	45.78^b^	50.11^a^	52.37^a^	0.79	0.05
a∗	6.08^ab^	6.81^a^	5.68^ab^	5.82^ab^	6.55^a^	4.33^b^	0.32	0.04
b∗	14.20^ab^	12.07^ab^	13.41^ab^	15.68^a^	15.72^a^	11.47^b^	0.43	0.03
TL (%)	2.69^b^	4.11^b^	5.76^ab^	2.85^b^	3.23^b^	7.87^a^	0.55	0.02
CL (%)	53.32^a^	50.79^a^	44.90^b^	48.67^ab^	48.29^ab^	48.54^ab^	0.93	0.04
Tenderness (N)	6.77	6.29	6.72	6.78	6.23	6.84	0.08	0.10

a,b, c Means within columns with different superscripts differ significantly (P<0.05), NEGCON; Negative control diet (0% additives), POSCON; positive control diet with 200g a-tocopherol acetate per ton; PPPM; Pomegranate peel powder meal at different inclusion levels of 2, 4, 6 and 8 g/kg; L∗= lightness; a∗ = blueness; b∗ yellowness; TL: Thawing loss: CL; Cooking loss; SEM; standard error of mean.

### 3.2 Shelf-life attributes of meat during storage

#### 3.2.1 Meat pH

As shown in Table 5, the supplementation of pomegranate peel powder meal (PPPM) had significant (p < 0.05) effect on the pH of meat on days 13 and 16 of storage. On the 13th day, NEGCON and PPPM_4_ meat samples had higher (P<0.05) pH values compared with the pH values recorded for other dietary treatments. On day 16, higher (P<0.05) pH values were recorded for NEGCON, PPPM_4_, PPPM_6_ and PPPM_8_ compared with the other dietary treatments.

**Table 5:** Effect of dietary pomegranate peel powder meal on breast meat pH during refrigerated storage.

Attributes	Dietary treatments	Days of storage							
		1	2	3	4	5	6	7	8
pH	NEGCON POSCON PPPM_2g/kg_ PPPM_4g/kg_ PPPM_6g/kg_ PPPM_8g/kg_ SEM P-value	5.67	6.08	5.87	5.81	5.86	5.75	5.91	5.84
		5.69	5.57	5.94	5.77	5.86	5.84	5.88	5.87
		5.65	5.78	5.79	5.67	5.79	5.84	5.77	5.83
		5.69	5.87	5.91	5.86	5.93	5.85	5.90	6.00
		5.64	5.87	5.84	5.71	5.86	5.83	5.81	5.88
		5.76	5.86	5.89	5.88	5.85	5.87	5.83	5.86
		0.02	0.02	0.01	0.01	0.02	0.02	0.01	0.02
		0.89	0.37	0.73	0.31	0.41	0.08	0.14	0.48
Attributes	Dietary treatments	Days of storage							
		9	10	11	12	13	14	15	16
pH	NEGCON POSCON PPPM_2g/kg_ PPPM_4g/kg_ PPPM_6g/kg_ PPPM_8g/kg_ SEM P-value	6.17	6.42	6.38	6.36	6.43^a^	6.38	6.42	6.40^a^
		6.06	6.00	6.00	6.40	6.12^b^	6.25	6.29	6.16^b^
		5.96	6.03	5.92	6.39	6.10^b^	6.17	6.25	6.03^b^
		6.83	6.78	6.75	6.49	6.63^a^	6.46	6.48	6.54^a^
		6.23	6.23	6.22	6.19	6.18^b^	6.29	6.32	6.43^a^
		6.19	6.30	6.29	6.15	6.25^ab^	6.14	6.36	6.36^a^
		0.05	0.05	0.06	0.06	0.04	0.05	0.05	0.05
		0.27	0.47	0.19	0.07	0.02	0.15	0.12	0.01

^a,b, c^Means within columns with different superscripts differ significantly (P<0.05), NEGCON; Negative control diet (0% additives), POSCON; positive control diet with 200g a-tocopherol acetate per ton; PPPM; Pomegranate peel powder meal at different inclusion levels of 2, 4, 6 and 8 g/kg; SEM; standard error of mean.

#### 3.2.2 Meat colour

There were significant (P<0.05) treatment effects on the Hunter Lab colour (L∗, a ∗, and b∗) traits of meat during the 16-d storage trial (Table 6). On day 3, the highest (P<0.05) L∗ value was recorded for PPPM_2_ and POSCON meat, while the PPPM_4_ group had the lowest (P<0.05) value for L∗. On day 9, L∗ value was highest in the PPPM_2_ and lowest in the PPPM_8_. Redness (a∗) was affected on days 5, 7, and 8 of storage. On day 5, PPPM_8_ meat had the highest (P<0.05) a∗ value, while PPPM_4_ had the lowest a∗. On day 7, redness was highest in PPPM_8_ and lowest in meat from the PPPM_4_ group. On day 8, higher a∗ values were recorded for NEGCON and PPPM_2_, while a∗ value was lowest in PPPM_6_. Yellowness (b∗) was affected on days 2, 5, 7, and 8. On day 2, a higher (P<0.05) b∗ value was recorded for POSCON meat, while PPPM_8_ and NEGCON meat had lower b∗ values. On day 5, meat from birds fed NEGCON, POSCON, and PPPM_6_ diets had the highest b∗ values, while those from PPPM_2_ and PPPM_8_ had the lowest b∗ values. Meat from all PPPM groups had the lowest (P<0.05) b∗ values on day 7, while the highest (P<0.05) b∗ values were recorded for in meat from the control groups. On day 8, b∗ was higher (P<0.05) in POSCON and PPPM_4_ samples, while the PPPM_2_ had lower values for b∗.

**Table 6:** Effect of dietary pomegranate peel powder meal on breast meat colour traits during refrigerated storage.

Attributes	Treatments	Days of storage							
		1	2	3	4	5	6	7	8
L∗	NEGCON POSCON PPPM_2g/kg_ PPPM_4g/kg_ PPPM_6g/kg_ PPPM_8g/kg_ SEM	51.14	50.32	47.02^ab^	46.61	49.27	48.83	49.07	47.86
		47.37	49.22	48.19^a^	46.63	48.18	47.04	48.48	48.74
		50.71	50.37	49.12^a^	48.77	48.23	49.18	48.06	48.81
		45.78	46.42	46.46^b^	45.24	49.19	46.41	49.49	48.86
		50.11	55.89	47.60^ab^	47.78	47.25	48.86	48.87	51.24
		52.37	50.54	47.21^ab^	45.42	47.08	46.49	46.69	47.87
		0.79	0.70	0.52	0.61	0.57	0.51	0.67	0.68
	P-value	0.56	0.79	0.01	0.52	0.12	0.57	0.36	0.54
a∗	NEGCON POSCON PPPM_2g/kg_ PPPM_4g/kg_ PPPM_6g/kg_ PPPM_8g/kg_ SEM	6.08	8.78	7.37	7.63	7.83^ab^	6.69	7.56^c^	6.87^a^
		6.81	9.23	7.26	7.83	7.61^ab^	7.43	7.25^c^	6.03^ab^
		5.68	7.35	7.34	7.90	7.47^ab^	7.32	8.28^b^	6.40^a^
		5.82	8.39	8.64	9.15	7.32^b^	8.84	5.69^e^	5.90^b^
		6.55	6.38	7.70	7.19	7.88^ab^	7.50	6.48^d^	4.52^c^
		4.34	8.48	7.38	9.57	8.13^a^	8.25	9.22^a^	5.97^b^
		0.32	0.56	0.36	0.40	0.36	0.40	0.34	0.31
	P-value	0.44	0.22	0.17	0.87	0.03	0.15	0.00	0.03
b∗	NEGCON POSCON PPPM_2g/kg_ PPPM_4g/kg_ PPPM_6g/kg_ PPPM_8g/kg_ SEM	14.20	16.87^c^	18.70	18.05	18.03^a^	18.93	18.26^ab^	17.66^b^
		12.07	20.14^a^	18.23	18.26	18.41^a^	17.87	19.49^a^	19.01^a^
		13.41	17.31^b^	16.53	16.04	16.76^c^	16.76	17.09^b^	16.04^c^
		15.68	17.06^b^	18.76	21.07	17.87^b^	19.64	17.65^b^	18.42^a^
		15.72	17.37^b^	17.81	19.19	18.47^a^	16.75	17.75^b^	17.82^b^
		11.47	16.95^c^	16.70	17.65	16.89^c^	16.39	17.88^b^	17.74^b^
		0.43	0.57	0.35	0.46	0.52	0.36	0.57	0.61
	P-value	0.23	0.05	0.84	0.51	0.02	0.14	0.02	0.05
Attributes	Treatments	Days of storage							

9	10	11	12	13	14	15	16
L∗	NEGCON POSCON PPPM_2g/kg_ PPPM_4g/kg_ PPPM_6g/kg_ PPPM_8g/kg_ SEM	44.00^ab^	43.88	41.99	41.99	42.57	42.55	40.93	42.25
		43.46^c^	43.08	41.70	41.29	41.58	41.29	40.84	41.16
		49.32^a^	47.46	45.43	46.42	45.42	46.42	49.05	47.62
		44.96^b^	43.11	41.32	40.38	40.43	40.38	41.13	40.63
		44.64^b^	45.25	43.08	44.13	43.47	44.13	47.56	45.45
		41.13^d^	41.57	40.00	40.14	41.19	40.14	41.99	42.54
		0.80	0.90	0.81	0.72	0.81	1.11	1.13	1.02
	P-value	0.01	0.31	0.46	0.90	0.59	0.17	0.08	0.26
a∗	NEGCON POSCON PPPM_2g/kg_ PPPM_4g/kg_ PPPM_6g/kg_ PPPM_8g/kg_ SEM	9.04	8.77	8.96	9.77	9.88	9.12	12.12	9.29
		8.66	8.51	8.27	7.68	7.99	8.87	8.85	9.75
		6.28	6.45	6.61	8.03	8.14	7.76	6.98	7.71
		7.71	7.66	8.03	8.44	9.01	9.23	9.63	9.12
		8.31	8.34	8.78	8.84	8.46	8.25	6.78	7.98
		10.79	10.51	9.77	10.94	10.97	11.54	10.92	10.65
		0.43	0.44	0.49	0.47	0.42	0.49	0.56	0.46
	P-value	0.12	0.53	0.07	0.83	0.67	0.32	0.22	0.24
b∗	NEGCON POSCON PPPM_2g/kg_ PPPM_4g/kg_ PPPM_6g/kg_ PPPM_8g/kg_ SEM	16.66	17.30	17.84	19.39	19.77	16.83	17.09	17.32
		20.45	20.82	20.17	17.74	17.07	19.66	19.08	20.18
		15.31	16.15	15.08	16.79	16.71	15.76	14.11	14.60
		18.95	17.83	19.26	19.95	19.56	19.89	19.96	20.18
		17.83	16.57	17.02	16.40	15.04	16.06	13.76	15.56
		16.91	16.30	15.52	16.69	18.49	19.57	19.20	19.12
		0.75	0.67	0.59	0.51	0.57	0.54	0.72	0.71
	P-value	0.47	0.12	0.84	0.43	0.35	0.16	0.09	0.10

^a,b, c^ Means within columns with different superscripts differ significantly (P<0.05), NEGCON; Negative control diet (0% additives), POSCON; positive control diet with 200g a-tocopherol acetate per ton; PPPM; Pomegranate peel powder meal at different inclusion levels of 2, 4, 6 and 8 g/kg; SEM; standard error of mean.

#### 3.2.3 Hue angle and saturation index (chroma)

Although there were no dietary treatment effects on the Hue angle, the saturation index was significantly (P<0.05) influenced on days 2, 5, 7, and 8 of storage (Table 7). On day 2, POSCON meat had the highest chroma compared with meat from other dietary treatments. On day 5, the highest chroma was recorded in meat from the NEGCON, POSCON, and PPPM_6_ groups compared with those from PPPM_2_ and PPPM_8_. On day 7, NEGCON, POSCON and PPPM_8_ had the highest chroma, whereas, PPPM_4_ and PPPM_6_ meat were the lowest in chroma. On day 8, chroma was highest in NEGCON, POSCON, PPPM_4_ and PPPM_8_ and lowest in PPPM_2_.

**Table 7:** Effect of dietary pomegranate peel powder meal on Hue angle and chroma of breast meat during refrigerated storage.

Attributes	Treatments	Days of storage							
		1	2	3	4	5	6	7	8
Hue angle	NEGCON POSCON PPPM_2g/kg_ PPPM_4g/kg_ PPPM_6g/kg_ PPPM_8g/kg_ SEM P-value	66.90	63.25	68.72	67.24	66.25	70.74	67.43	68.24
		60.42	65.34	68.32	66.71	67.45	67.60	69.55	72.38
		67.23	67.58	66.11	64.13	66.01	66.54	64.51	67.95
		69.90	63.59	65.45	66.50	67.96	65.93	71.49	72.18
		67.31	69.55	66.36	69.21	66.74	65.56	69.84	75.69
		68.99	63.58	65.90	61.63	66.32	64.32	62.89	71.38
		1.05	1.04	0.98	1.03	0.99	1.06	0.83	0.87
		0.44	0.44	0.41	0.37	0.10	0.37	0.23	0.07
Chroma	NEGCON POSCON PPPM_2g/kg_ PPPM_4g/kg_ PPPM_6g/kg_ PPPM_8g/kg_ SEM P-value	15.47	18.89^b^	20.14	19.60	19.78^a^	20.11	19.79^a^	18.97^a^
		13.94	22.22^a^	19.69	19.91	19.94^a^	19.42	20.86^a^	19.95^a^
		14.63	19.12^b^	18.09	17.92	18.36^b^	18.31	19.06^ab^	17.29^b^
		16.77	19.19^b^	20.76	23.00	19.37^ab^	21.64	18.57^b^	19.44^a^
		17.10	18.61^b^	19.47	20.62	20.14^a^	18.39	18.93^b^	18.39^ab^
		12.28	19.12^b^	18.29	20.18	18.78^b^	18.43	20.13^a^	18.78^a^
		0.46	0.58	0.36	0.49	0.53	0.39	0.61	0.62
		0.10	0.00	0.50	0.29	0.01	0.61	0.01	0.02
Hue angle		Days of storage							

9	10	11	12	13	14	15	16
	NEGCON POSCON PPPM_2g/kg_ PPPM_4g/kg_ PPPM_6g/kg_ PPPM_8g/kg_ SEM P-value	60.64	62.87	63.98	62.88	62.95	60.90	54.54	61.39
		66.77	67.62	67.56	66.21	64.29	65.54	64.69	64.36
		67.03	66.47	66.72	64.94	64.19	64.58	61.44	61.19
		67.48	66.61	67.09	67.19	65.02	64.96	64.32	65.31
		65.68	63.63	62.68	62.35	61.52	63.87	64.31	62.72
		57.34	57.26	57.30	56.98	59.41	59.48	60.29	60.91
		1.27	1.45	1.38	1.16	1.15	1.34	1.63	1.17
		0.11	0.13	0.14	0.56	0.65	0.29	0.39	0.71
Chroma	NEGCON POSCON PPPM_2g/kg_ PPPM_4g/kg_ PPPM_6g/kg_ PPPM_8g/kg_ SEM P-value	19.14	19.57	20.12	21.79	22.21	19.25	21.08	19.73
		22.25	22.53	21.82	19.47	18.98	21.68	21.22	22.58
		16.67	17.65	16.59	18.66	18.64	17.62	15.86	16.56
		20.51	19.49	20.95	21.67	21.58	21.97	22.22	22.22
		19.72	18.61	19.16	18.74	17.34	18.27	15.58	17.56
		20.07	19.41	18.50	19.98	21.53	22.76	22.12	21.91
		0.76	0.65	0.63	0.57	0.59	0.58	0.76	0.74
		0.49	0.23	0.44	0.72	0.68	0.13	0.13	0.10

^a,b, c^ Means within columns with different superscripts differ significantly (P<0.05), NEGCON; Negative control diet (0% additives), POSCON; positive control diet with 200g a-tocopherol acetate per ton; PPPM; Pomegranate peel powder meal at different inclusion levels of 2, 4, 6 and 8 g/kg; SEM; standard error of mean.

#### 3.2.4 Effect of storage duration on meat quality

Table 8 shows the effects of storage duration on the shelf life attributes of meat during the 16-d storage duration. The lowest pH values were recorded on days 2 and 4, while pH values were highest on other storage days. Storage duration affected mean L∗ values, with meat being lighter on day 5 and darker on the 9th day (Table 8). Storage duration also affected a∗ and b∗ values. The lowest a∗ value was recorded on day 7 and the highest on day 15. The lowest b∗ value was recorded on day 1, while the highest values were recorded on other days of storage. Storage duration also had a significant (P<0.05) effect on the Hue angle and Chroma. The hue angle was highest on day 8 and lowest on the 15th day, whereas Chroma was lowest on the first day and highest on other storage days.

**Table 8:** Effect of storage duration on the shelf-life attributes of breast meat.

Storage days	Shelf-life attributes					
	pH	L∗	a∗	b∗	HA	Chroma
1	5.68^b^	49.58^ab^	5.88^d^	13.7^b^	66.79^bc^	15.03^b^
2	5.84^b^	50.46^a^	8.10^abc^	17.62^a^	65.48^bcde^	19.53^a^
3	5.87^b^	47.60^b^	7.62^bc^	17.79^a^	66.81^bc^	19.41^a^
4	5.78^b^	46.74^bc^	8.21^abc^	18.38^a^	65.90^bcd^	20.21^a^
5	5.86^b^	48.20^ab^	7.71^abc^	17.74^a^	66.79^bc^	19.39^a^
6	5.83^b^	47.80^ab^	7.67^abc^	17.72^a^	66.78^bc^	19.38^a^
7	5.85^b^	48.44^ab^	7.41^c^	18.02^a^	67.62^b^	19.56^a^
8	5.88^b^	48.89^ab^	5.95^d^	17.78^a^	71.30^a^	18.80^a^
9	6.24^a^	44.59^cd^	8.47^abc^	17.69^a^	64.16^bcde^	20.07^a^
10	6.29^a^	44.06^d^	8.37^abc^	17.49^a^	64.08^bcde^	19.73^a^
11	6.26^a^	42.25^b^	8.40^abc^	17.48^a^	64.22^bcde^	19.54^a^
12	6.33^a^	42.39^b^	8.95^abc^	17.83^a^	63.43^cde^	20.05^a^
13	6.29^a^	42.44^b^	9.08^ab^	17.77^a^	62.89^cde^	20.05^a^
14	6.28^a^	42.49^b^	9.13^ab^	17.96^a^	63.22^cde^	20.26^a^
15	6.35^a^	43.58^b^	9.21^a^	17.20^a^	61.59^e^	19.68^a^
16	6.32^a^	43.28^b^	9.08^ab^	17.83^a^	62.65^de^	20.09^a^
SEM	0.03	0.35	0.15	0.18	0.75	0.20
P-value	0.00	0.00	0.00	0.01	0.00	0.00

^a,b, c^ Means within columns with different superscripts differ significantly (P<0.05), L∗; Lightness; a∗; redness; b∗; yellowness; HA; Hue angle; SEM; standard error of mean.

#### 3.2.5 Antioxidant capacity and enzyme activities of broiler meat

**Figure 1:**
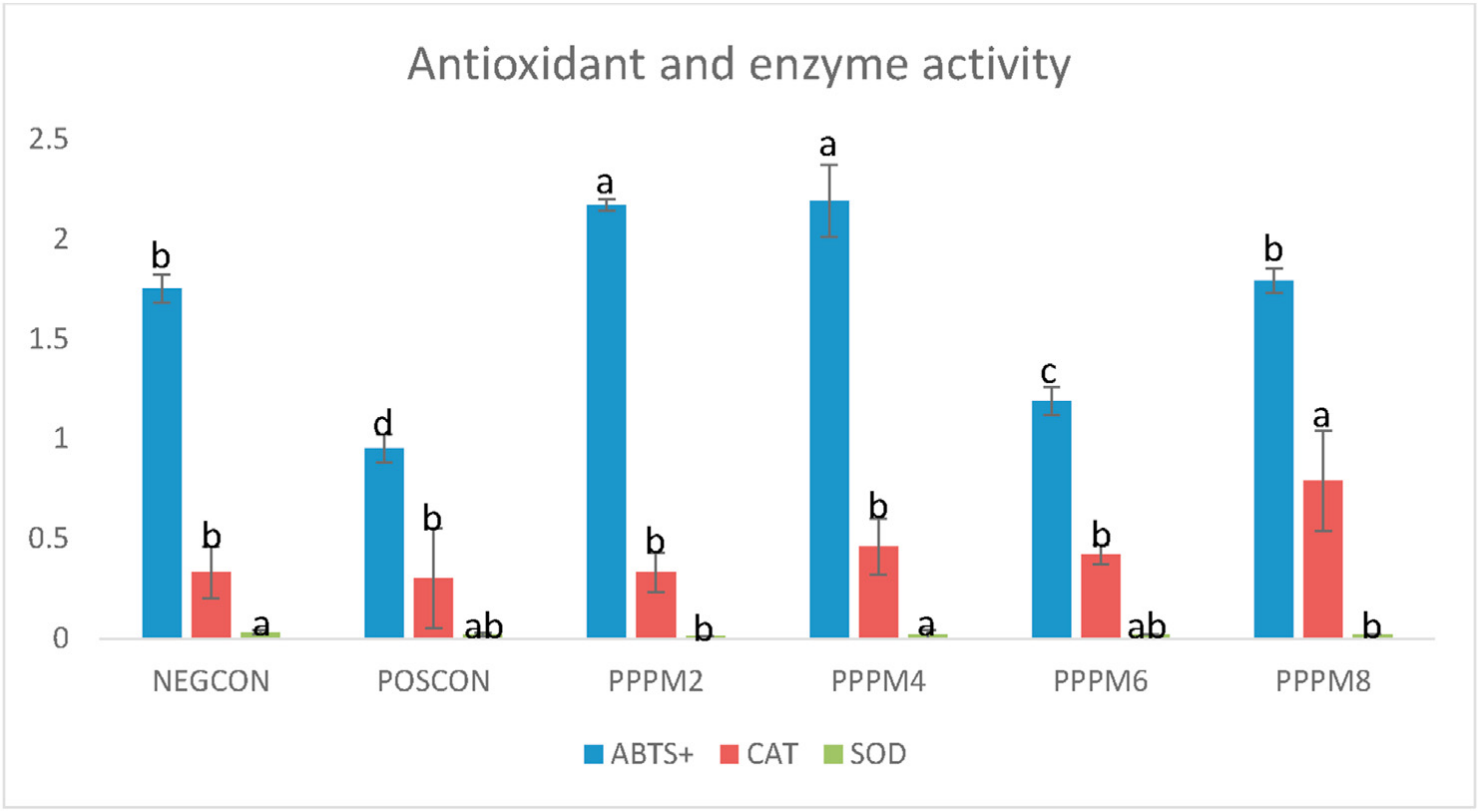
Effect of pomegranate peel powder meal (PPPM) supplementation on antioxidant and enzyme activities of broiler meat under refrigerated storage for 16-days.

At the end of the 16-d storage, the highest ABTS^+^ scavenging ability was recorded in meat from the PPPM_2_ and PPPM_4_ groups, whereas POSCON meat had the lowest ABTS^+^ value (Figure 1). Based on day effect, the highest ABTS^+^ values were recorded in PPPM_2_ on day 1 of storage, while the highest ABTS^+^ values were recorded for PPPM_2_ and PPPM_4_ on day 16 (Table 9). At the end of the 16-d storage, catalase (CAT) activity was highest in PPM_8_ compared with the other dietary treatments (Figure 1). Based on day effect, CAT was highest (P<0.05) in PPPM_8_ meat on days 1 and 16 of storage, and lowest in the PPPM_2_, POSCON, and PPPM_6_. At the end of the 16-d storage, superoxide dismutase (SOD) activity was highest in NEGCON and PPPM_4_ samples and lowest in PPPM_2_ and PPPM_8_ (Figure 1). Based on day effect, SOD activity was undetected in the meat of birds fed 2 g/kg and 4 g/kg PPPM on day 1 of storage, whereas meat from birds fed other treatment diets had similar SOD activity. On day 16th of storage, SOD activity was not significantly influenced by dietary treatments (Table 9).

**Table 9:** Effect of dietary pomegranate peel powder meal on antioxidant and enzyme activity of breast meat during refrigerated storage.

Attributes	Dietary treatments	Days of storage	
		Day 1	Day 16
SOD (U/mg protein)	NEGCON POSCON PPPM2g/kg PPPM4g/kg PPPM6g/kg PPPM8g/kg SEM P-value	0.01^a^	0.04
		0.01^a^	0.02
		0.00^b^	0.02
		0.00^b^	0.03
		0.01^a^	0.02
		0.01^a^	0.02
		0.00	0.01
		0.00	0.81
CAT (U/mg protein)	NEGCON POSCON PPPM2g/kg PPPM4g/kg PPPM6g/kg PPPM8g/kg SEM P-value	0.10^cd^	0.56^b^
		0.15^c^	0.45^c^
		0.07^d^	0.59^b^
		0.35^b^	0.56^b^
		0.37^b^	0.46^c^
		0.70^a^	0.87^a^
		0.01	0.02
		0.02	0.03
ABTS	NEGCON POSCON PPPM2g/kg PPPM4g/kg PPPM6g/kg PPPM8g/kg SEM P-value	1.52^c^	1.97^bc^
		0.49^e^	1.41^c^
		2.15^a^	2.19^ab^
		1.75^b^	2.64^a^
		0.60^e^	1.78^bc^
		1.32^d^	2.26^b^
		0.05	0.19
		0.05	0.04

^a,b, c^Means within columns with different superscripts differ significantly (P<0.05), PPPM: Pomegranate peel powder; NEGCON; Negative control diet (0% additives), POSCON; positive control diet with 200g a-tocopherol acetate per ton; PPPM; Pomegranate peel powder meal at different inclusion levels of 2, 4, 6 and 8 g/kg; SOD: Superoxide dismutase; CAT: Catalase; ABTS; [(2, 2-azinobis (3ethylbenzothiazoline-6 sulfonic acid))] scavenging activity; SEM; standard error of mean.

## 4 Discussion

### 4.1 Physico-chemical attributes of meat

Having a detailed knowledge of the postmortem physicochemical attributes (e.g., pH, colour, etc.) of poultry meat and the quality under storage could help ascertain its post-slaughter quality (Janisch et al., 2012). Postmortem pH (pHu) is the primary determinant of the colour, tenderness, cooking loss, water holding capacity, and other sensory attributes of meat (Shang et al., 2014). A typical ultimate pHu range for standard broiler meat has been reported as 5.6–5.9 (Garcia et al., 2010) or 6.1 (Mikulski et al. 2010). Although pHu was not significant in this study, the values (5.64–5.76) were within the reported range of pHu for typical broiler meat (Table 4). As also shown in Table 4, meat from birds fed the PPPM_2_, PPPM_6_ and PPPM_8_ diets had the highest L∗ value. Mancini and Hunt (2005) had earlier reported a high correlation between muscle pH and colour. Usually, lighter meat has lower pH, and when the L∗ value exceeds 56 or 59 (Woefel et al., 2002; Petracci et al., 2004), the meat is considered to be pale, soft, and exudative (PSE), whereas, darker meat have higher pH values, and in extreme cases are characterized as dry, firm and dark meat (DFD) (Fletcher, 2002). However, there are inconsistencies in the literature on typical L∗ and pH values for standard broiler chicken meat. (Qiao et al., 2001) reported that typical broiler meat has L∗ range of 45.1–55.1, whereas other reports (Woefel et al., 2002; Petracci et al., 2004) reported 47 ≤ L∗≤53 as the L∗ values for standard meat depending on the animal and management-related factors that affect colour. The L∗ values were also within typical L∗ values for standard broiler meat.

Thawing loss had its highest value in the meat of birds fed 8 g/kg PPPM diet, whereas cooking loss was significantly decreased at 2 g/kg PPPM inclusion (Table 4). An earlier report (Mahmmod, 2014) showed that pomegranate peel extract has a high water-binding capacity, which improves the water holding capacity of meat, thereby reducing the thaw and cooking losses. The exact reason for the high thawing loss in PPPM_8_ samples is unknown, considering its ultimate pH and the pH values during the 16-d refrigerated storage. An increase in percentage thawing loss is due to the low water-holding capacity of meat, which may be due to decreased pH and rigor state and denaturation of water-binding proteins (Savelle et al., 2005; Hannula and Poulanne, 2004; Leygonie et al., 2012). This outcome may also be due to other factors such as handling, packaging, differences in aging, storage, freezing, and thawing conditions, all of which affect meat quality (Adzitey, 2011; Tougan et al., 2013).

### 4.2 Meat pH

At slaughter, the pH of meat is approximately neutral. Following slaughter however, the permeability of cell membrane changes, resulting in low water holding capacity of the muscles (Huff-Lonergan and Lonergan, 2005). This change is attributed to the accumulation of a large amount of lactic acid in the muscles (due to the breakdown of glycogen), coupled with the stoppage of blood flow, which induces cellular hypoxia and reduce the pH of the meat to an ultimate pH (pH_u_) value of 5.4–5.8 (Lambert et al., 2001). However, this decline in pH needs to be limited to ensure stability during storage (Jalosinska and Wilczak, 2009) and enhance the functional attributes of meat (Jlali et al., 2012). The pH of meat is an essential factor that influences the colour, tenderness, cooking loss, shelf-life, and other physicochemical properties (Shang et al., 2014). When meat pH rapidly declines, the tendency for it to be pale and have low water holding capacity increases (Lipinski et al. 2019). From the results in this study (Table 5), dietary treatments significantly influenced the pH of meat on days 13 and 16 of storage. On the 13th day, the pH values of meat from birds fed PPPM_2_, PPPM_6_, and POSCON diets were within the range of 5.9–6.2 reported by Zywica et al. (2011) as pH for normal broiler meat, whereas, on day 16, only those from 2 g/kg PPPM and POSCON dietary groups were within this normal pH range for broiler meat. According to Zywica et al. (2011), pH values below 5.7 and higher than 6.4 are pointers to PSE (pale-soft and exudative) and DFD (dry, firm, and dark) conditions in meat.

Interestingly, on both days 13 and 16 of storage, low pH was recorded in PPPM_2,_ PPPM_6_ and POSCON meat samples. This result align with the reports of Jalosinska and Wilczak (2009), which showed that reduced pH during extended storage, indicates slow/reduced meat spoilage by microbial agents. Similarly, Ahmed et al. (2015) reported that after 14 days of storage, meat from broiler birds fed dietary pomegranate by-products had lower pH values than the control. Pomegranate peel (powder and extracts) can retard meat spoilage due to bacterial activity linked to its rich content of tannins and phenolic acid (Devatkal et al., 2013). Antioxidants like tocopherol acetate also slow down the microbial spoilage of stored meat (Li and Liu, 2012).

### 4.3 Meat colour

Broiler meat possesses intrinsic attributes like appearance, texture, juiciness, and flavor (Petracci and Beza, 2011). Thus, meat colour is the most crucial factor determining the procurement of broiler meat by consumers (Werner et al., 2009). Hunter Lab colour (L∗, a ∗, and b∗) traits were affected by pomegranate peel powder meal (PPPM) supplementation, as shown in Table 6. The mean L∗ values ranged from 45.78-52.37 at 24 h postmortem and were within the typical L∗ range for standard broiler meat (Qiao et al., 2001; Garcia et al., 2010). The results in Table 6 showed that on day 3 of refrigerated storage, meat from birds fed the POSCON and PPPM_2_ diets had the highest L∗ values compared to that from birds fed the PPPM_4_ diet, while on day 9, L∗ had its highest value in meat from the PPPM_2_ group compared to that from birds fed POSCON, PPPM_4_, PPPM_6_ and PPPM_8_ diets. Werner et al. (2011) had reported that changes in postmortem meat colour are due to mitochondria-antioxidant enzyme interactions, which impact the oxidative state of the tissue. Higher a∗ values were recorded for the 8 g/kg PPPM dietary groups on days 5 and 7 of refrigerated storage, and for the NEGCON and 2 g/kg PPPM groups on day 8 (Table 6). Meat from 4 g/kg, 6 g/kg and 8 g/kg PPPM groups competed favourably with the POSCON group in reflecting high b∗ values on days 5, 7, and 8 of storage (Table 6). High b∗ values have been associated with beta carotene content of broiler diets due to the inclusion of natural antioxidant compounds (Moyo et al., 2011). A high chroma value is an indication that the meat was more saturated and intense. Medicinal plants have been reported to improve chroma values due to their tannin content, which possesses colour-stabilizing antioxidant effects (Moyo et al., 2011). Pomegranate peel is a rich source of hydrolysable tannins such as ellagitannin, gallotannins, and gallagyl esters like punicalagin, punocaliin, and pedunculagin (Naveena et al., 2008; Madrigal-Carballo et al., 2009), all of which enhances meat quality. Similarly, Tocopherol acetate has the potential to enhance meat colour due to its ability to inhibit the oxidation of myoglobin and/or oxymyoglobin to metmyoglobin (Karami et al., 2010).

### 4.4 Total antioxidant capacity and enzyme activity of broiler meat

Medicinal plants possess antioxidant effects due to the presence of polyphenols. Polyphenols can remove free radicals, chelate metal catalysts, stimulate antioxidant enzymes, reduce α-tocopherol radicals, and prevent oxidases (Shad et al., 2014; Nimse and Pal, 2015). In the present study, dietary PPPM improved the ability of samples to scavenge the free radical ABTS (Table 9). Meat from 2 g/kg PPPM-treated birds had the highest ATBS scavenging ability on day 1, whereas on day 16, the highest capacity to scavenge ABTS was exhibited by 4 g/kg PPPM. It was evident that the capacity of meat samples to eliminate the free radical ABTS increased remarkably during the 16-d duration of storage compared with the 24-hour postmortem (i.e., day 1). In terms of overall storage effect, total ABTS scavenging ability was highest in meat from birds fed 2 g/kg and 4 g/kg PPPM diets and lowest in the POSCON (Figure 1).

Poultry meat contains antioxidant compounds that influence its quality. Generally, antioxidants play an essential role in maintaining the innate defenses of cells and tissues, particularly as any debilitation in the activity of these cells causes a dysfunction (Ibrahim et al., 2019). It is important to note that the structure of meat proteins and lipids changes under storage conditions, especially as oxidation progresses. These changes, coupled with the loss of moisture from the meat as a result of evaporation (Ibrahim et al., 2019), affect the meat's stability during storage. Interestingly, the supplementation of pomegranate peel has been shown to improve broiler meat's oxidative stability, particularly under storage conditions. Suresh et al. (2010) reported significant improvement in the antioxidant properties of goat meat stored at 4 °C when pomegranate peel powder was included in the diet of goat kids, alongside pomegranate seed powder, kinnow rind powder, and salt. The authors concluded that pomegranate peel powder could reduce auto-oxidation and salt-induced lipid oxidation in raw ground goat meat.

Verma et al. (2009) reported that medicinal plants contain antioxidant compounds that have direct and indirect capacities of minimizing or inhibiting lipid oxidation in tissues. They either directly scavenge for free radical species, or indirectly improve the cell's innate defense mechanisms, by activating the activities of antioxidant enzymes such as SOD, CAT, etc. Superoxide dismutase (SOD) and catalase (CAT) are key hepatic antioxidant enzymes that catalytically scavenge free radicals and other ROS, thereby conferring endogenous protection on biological systems against oxidative stress (Saleh et al., 2017a). These endogenous antioxidant enzymes help to maintain the health of poultry birds, as well as the physiology of antioxidative systems (Ajuwon et al., 2016). At the cellular level, these enzymes have different localizations, and they serve as first-line antioxidant defense mechanisms that protect mammalian cells against the deleterious effects of ROS (Halliwell and Gutteridge, 2007; Ajuwon et al., 2016). In this study, SOD activity was undetected in the meat of birds fed 2 g/kg and 4 g/kg PPPM on day 1 of the refrigerated storage, whereas meat from birds fed other treatment diets had similar SOD activity (Table 9). On the 16th day of storage, SOD activity was unaffected by dietary treatments. Interestingly, there was a marked increase in CAT activity observed in the meat of the 8 g/kg PPPM-treated birds compared with the control groups (Table 9). Also, the total CAT activity was highest in 8 g/kg PPPM compared with those of the other dietary treatments (Figure 1). The total SOD activity was highest in meat from the NEGCON and PPPM_4_ and lowest in PPPM_2_ and PPPM_8_ (Figure 1).

According to Ajuwon et al. (2016), CAT degrades the hydrogen peroxide (H_2_0_2_) produced by SOD to oxygen and water. The detoxification of the hydroxyl radicals confers antioxidant protective capacity on biological systems. An earlier report (Ajuwon et al., 2013) had shown that an increase in CAT activity in the erythrocytes of tert-butyl-hydroperoxide-treated rats was due to the formation of H_2_0_2_ by SOD and/or up-regulation of expression of the gene encoding for CAT. An increase in SOD and CAT activity in broiler meat/blood plasma is an indication that these enzymes could act faster to remove free radicals in broiler meat and blood samples (Akbarian et al. 2015). Momenah (2017) reported a significant increase in blood plasma CAT activity in rabbits fed with pomegranate peel powder extract. This upturn was due to the increased hepatocellular activities in rabbits exposed to the pomegranate peel powder extract, as opposed to those that received the control diet. SOD has been reported to be the main enzyme involved in the detoxification of ROS and is responsible for catalyzing the dis-mutation of superoxide anions to oxygen and H_2_0_2_ (Pagmantidis et al., 2005). The non-significant effects of SOD activity in breast meat of broiler birds fed diets supplemented with PPPM was not anticipated. Also, the reason for the non-detection of SOD activity in the breast meat of the 2 g/kg and 4 g/kg PPPM-treated birds on day 1 of storage are not precisely known. However, just like the results obtained in this study, Saleh *et al.* (2017a) observed that dietary inclusion of either pomegranate peel powder, pomegranate peel extract, or and α-tocopherol did not have a significant effect on SOD activity in the blood plasma of broiler birds.

## 5 Conclusion

The results obtained in this study indicate that 2 g/kg supplementation of pomegranate peel powder meal significantly improved the water-binding capacity of broiler breast meat, owing to the reduced cooking loss of the meat, and meat from the PPPM_2_ (2 g/kg) group had the highest ability to scavenge ABTS.

## Declarations

### Author contribution statement

Eunice A. Akuru: Conceived and designed the experiments; Performed the experiments; Analyzed and interpreted the data; Wrote the paper.

Chika E. Oyeagu: Performed the experiments; Analyzed and interpreted the data; Wrote the paper.

Thando C. Mpendulo: Performed the experiments; Wrote the paper.

Fanie Rautenbach, Oluwafemi O. Oguntibeju: Contributed reagents, materials, analysis tools or data; Wrote the paper.

### Funding statement

The authors are grateful to the NRF-TWAS African Renaissance Doctoral fellowship (Grant number: 110899) for funding the research of the first author. Prof. OO Oguntibeju also received funding from the 10.13039/501100004512Cape Peninsula University of Technology to support the work (CPUT-RJ23).

### Data availability statement

Data included in article/supplementary material/referenced in article.

### Declaration of interests statement

The authors declare no conflict of interest.

### Additional information

No additional information is available for this paper.

## Appendix A Supplementary data

Updated data for reviewed meat manuscript_Heliyon
